# Easing genomic surveillance: A comprehensive performance evaluation of long-read assemblers across multi-strain mixture data of HIV-1 and Other pathogenic viruses for constructing a user-friendly bioinformatic pipeline

**DOI:** 10.12688/f1000research.149577.1

**Published:** 2024-05-31

**Authors:** Sara Wattanasombat, Siripong Tongjai

**Affiliations:** 1 Department of Microbiology, Faculty of Medicine, Chiang Mai University, Chiang Mai, 50200, Thailand

**Keywords:** HIV, Virus, Infectious Diseases, NGS, Single-molecule sequencing, Haplotype reconstruction, Genome assembly, Genomic surveillance

## Abstract

**Background:**

Determining the appropriate computational requirements and software performance is essential for efficient genomic surveillance. The lack of standardized benchmarking complicates software selection, especially with limited resources.

**Methods:**

We developed a containerized benchmarking pipeline to evaluate seven long-read assemblers—Canu, GoldRush, MetaFlye, Strainline, HaploDMF, iGDA, and RVHaplo—for viral haplotype reconstruction, using both simulated and experimental Oxford Nanopore sequencing data of HIV-1 and other viruses. Benchmarking was conducted on three computational systems to assess each assembler’s performance, utilizing QUAST and BLASTN for quality assessment.

**Results:**

Our findings show that assembler choice significantly impacts assembly time, with CPU and memory usage having minimal effect. Assembler selection also influences the size of the contigs, with a minimum read length of 2,000 nucleotides required for quality assembly. A 4,000-nucleotide read length improves quality further. Canu was efficient among *de novo* assemblers but not suitable for multi-strain mixtures, while GoldRush produced only consensus assemblies. Strainline and MetaFlye were suitable for metagenomic sequencing data, with Strainline requiring high memory and MetaFlye operable on low-specification machines. Among reference-based assemblers, iGDA had high error rates, RVHaplo showed the best runtime and accuracy but became ineffective with similar sequences, and HaploDMF, utilizing machine learning, had fewer errors with a slightly longer runtime.

**Conclusions:**

The HIV-64148 pipeline, containerized using Docker, facilitates easy deployment and offers flexibility to select from a range of assemblers to match computational systems or study requirements. This tool aids in genome assembly and provides valuable information on HIV-1 sequences, enhancing viral evolution monitoring and understanding.

## Introduction

In 2020, UNAIDS has re-established the 95-95-95 targets to end the HIV/AIDS epidemic, aiming for 95% of people to know their HIV status, receive treatment, and achieve viral suppression by 2025. ^1^^–^^3^ While efforts to combat HIV-1 focus on treatment and prevention, HIV-1 genomic surveillance plays a crucial role. ^4^ It offers essential data for evidence-based strategies in HIV prevention, testing, treatment, and care. ^5^^,^^6^ The genomic surveillance protocol should entail simple and swift sample preparations and sequencing, facilitated by Oxford Nanopore Sequencing Technology. ^7^^–^^10^ Additionally, it should feature straightforward bioinformatic analysis pipelines and provide comprehensive reports enriched with reliable HIV-1 information. This adaptable protocol can utilize various computational resources, contributing significantly to global efforts against HIV/AIDS.

The lack of a proofreading mechanism in HIV-1 reverse transcriptase leads to a high mutation rate, potentially resulting in immune-evading variants or drug-resistant strains, posing challenges in patient care and virus transmission control strains. ^11^^–^^16^ Various patterns of intra-host multi-strain HIV infection, including dual, co-, super, and triple infection, have been observed. ^17^ Multiple HIV infections significantly contribute to the emergence of novel and more infectious HIV-1 recombinants, impacting viral fitness and increasing inter-subtype recombinants’ prevalence. ^18^^,^^19^ Some novel HIV-1 recombinants may be undetectable, especially in medically suppressed individuals. ^20^^,^^21^ Therefore, HIV-1 quasispecies profiling holds value for understanding viral population dynamics, particularly newly emerging recombinants. ^22^^–^^24^ Developing an effective monitoring protocol focusing on the viral genomic level is crucial for understanding HIV-1 dynamics within the host and devising better treatment and prevention strategies. ^21^^,^^25^

Recently, Next Generation Sequencing (NGS) technologies have become crucial in viral genome analysis. The Sequencing by Synthesis (SBS) approach enables the detection of single nucleotide variants (SNVs) and some alterations in viral genomes. ^26^ However, SBS technology faces limitations in accurately reconstructing viral haplotypes and detecting low-abundance haplotypes for quasispecies analysis. It is also inadequate for examining complex viral sequences such as HIV-1’s long terminal repeats (LTRs) or large deletions or insertions in the HIV envelope glycoprotein gene. Furthermore, SBS technology is not well-suited for analyzing phased mutations in the viral genome. ^20^^,^^26^

Single-molecule sequencing (SMS) technology, including Oxford Nanopore Technology (ONT) and PacBio SMRT technology, enhances viral quasispecies analysis by generating longer genomic reads, ^27^^–^^29^ enabling near-complete viral genome reconstruction with high accuracy (97–99% identity). ^17^ ONT’s recent advancements allow direct RNA sequencing, reducing bias from PCR or cDNA synthesis. ^24^ However, SMS technology has a high raw read sequencing error rate exceeding 10%, which can be mitigated with various long-read error correction methods. ^30^^–^^34^ ONT R10.3 chemistry sequencing of the HIV-1 genome yields raw reads with a 5 to 12% error rate, while ONT’s unique molecular identifier (UMI) method achieves a single molecule consensus accuracy of up to 99.9995%. ^35^^,^^36^ PacBio SMRT has an error rate of 13 to 15%, but its circular consensus sequencing (CCS) approach provides consensus reads with approximately 99.999% accuracy. ^35^^,^^36^ Data quality from SMS technology can be improved by implementing quality control measures, optimizing coverage thresholds, and validating variant calls to ensure the reliable detection of minor mutations despite residual errors. ^34^^,^^37^^,^^38^

Two categories of HIV-1 haplotype detection for SMS data are reference-based and *de novo* approaches. ^39^ The reference-based methods, though generally accurate, may introduce bias without suitable reference sequences. ^26^ Using a single reference genome can miscluster reads from different viral haplotypes. For example, RVHaplo’s hierarchical clustering with a reference genome may inaccurately group reads from different haplotypes, ^40^ affecting characterization of novel or rare haplotypes. Canu and GoldRush, considered general-purpose *de novo* assemblers, may overlook genomic reads with lower coverage. ^41^ Conversely, MetaFlye and Strainline are strain-aware *de novo* assemblers, recognizing strain differences within a sample. ^28^^,^^42^ HaploDMF, iGDA, and RVHaplo are reference-based assemblers designed for multi-strain mixtures. ^43^^–^^45^ iGDA and RVHaplo employ similar clustering approaches, while HaploDMF uses deep matrix factorization for contig extension. ^43^^,^^46^ Despite numerous assembly software options tailored for SMS data, ^28^^,^^42^^–^^45^^,^^47^^,^^48^ selecting the best tool for haplotype reconstruction and HIV-1 quasispecies analysis is challenging due to a lack of systematic studies. Furthermore, the absence of standardized benchmarking across different software and computing environments complicates the selection process, especially with limited computational resources. ^49^^,^^50^

In this study, we demonstrated that Strainline and MetaFlye excelled at haplotype reconstruction, although Strainline required more memory. Conversely, Canu performed poorly when distinguishing sequences in multi-strain mixtures, while GoldRush only yielded consensus assemblies. iGDA exhibited high error rates, whereas RVHaplo showed superior runtime and accuracy. HaploDMF offered improved accuracy despite a longer runtime. Additionally, a containerized pipeline, named HIV-64148, was developed to provide publicly accessible and user-friendly tool for genomic surveillance of HIV-1.

## Methods

### Benchmarking pipeline

**Figure 1.**
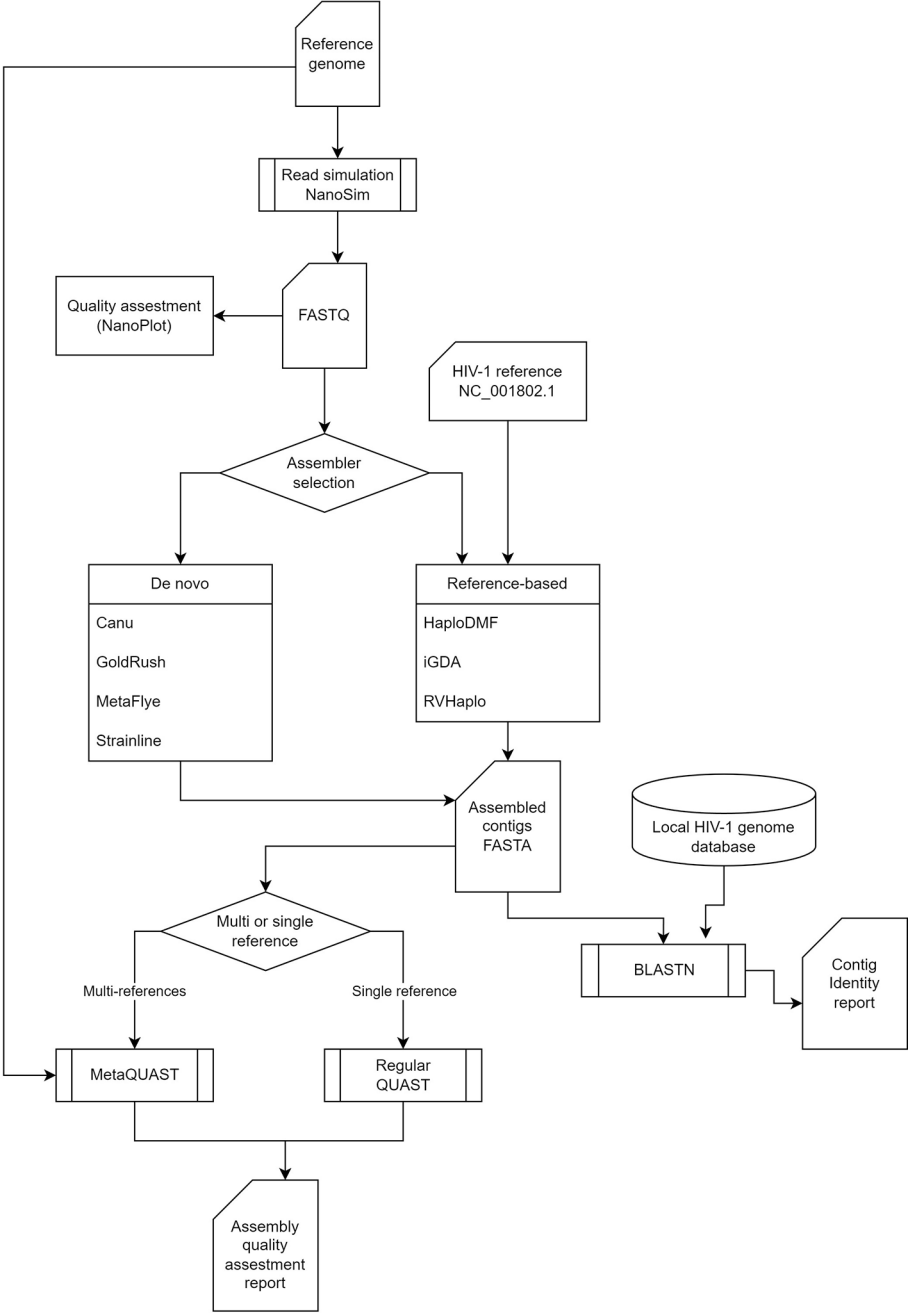
The HIV-64148 benchmarking pipeline. The pipeline begins with a read quality control analysis of long-read FASTQ files, followed by an assembler, which can be either *de novo* or reference-based. Finally, the pipeline includes HIV-1 subtyping to classify the HIV-1 strains present in the data.

The benchmarking pipeline ( Figure 1) started with an assessment of the quality of all long-read FASTQ inputs using NanoPlot ^51^ to ensure data conformity, particularly regarding the median read length. This study evaluates the performance and accuracy of seven assemblers on three different computational systems. Subsequently, the quality and accuracy of each assembler’s output are assessed using QUAST. Additionally, sequence similarity and HIV-1 subtype of all assembled contigs are investigated. The benchmarking pipeline is containerized, enabling its execution on any cloud or non-cloud environment ^52^ supporting either Docker or Singularity.

### Long-read assemblers

This study aimed to assess the accuracy, performance, and computational efficacy of seven candidate long-read assemblers, categorized into two approaches for haplotype reconstruction: (1) *de novo* long-read assemblers, which include Canu, ^47^ Goldrush, ^48^ MetaFlye, ^42^ and Strainline, ^28^ and (2) reference-based long-read assemblers, which comprise HaploDMF, ^43^ iGDA, ^44^ and RVHaplo. ^45^

The assembly software, including Canu, MetaFlye, and iGDA, was installed using the Micromamba package manager, which utilized recipes from the conda-forge ( https://anaconda.org/conda-forge) and bioconda channels ( https://anaconda.org/bioconda). An additional channel, zhixingfeng ( https://anaconda.org/zhixingfeng), was used specifically for iGDA as per the installation guidelines. Each tool was set up in separate environments to adhere to the developer’s specified version of dependencies.

For HaploDMF, RVHaplo, and Strainline, specifications were not available in Micromamba, necessitating manual installation. The source codes for HaploDMF ( https://github.com/dhcai21/HaploDMF) and RVHaplo ( https://github.com/dhcai21/RVHaplo) were downloaded from their respective GitHub repositories, with dependencies installed via Micromamba using environment specification files provided by the developers. For Strainline, the source code was sourced from its GitHub repository ( https://github.com/HaploKit/Strainline). Dependencies were managed and installed with Micromamba, except for Daccord ( https://github.com/gt1/daccord) and Metabat2 ( https://bitbucket.org/berkeleylab/metabat/src/master/), which were obtained by downloading the latest releases from the developers’ websites. Licensing for these tools varies: Strainline, GoldRush, HaploDMF, and RVHaplo are licensed under GPL3.0, Canu and iGDA under GPL2.0, and MetaFlye under the BSD-3-Clause license. Table 1 provides an overview of all candidate long-read assemblers.

**Table 1.** An overview of long read assemblers.

Assembler	Class	Type	Error handling	Haplotype reconstruction	Frequency estimation	Programming language
**Canu** ^**47**^	Graph-based (general assembler)	*de novo*	Two filtering steps, global filter to find correction evidence and local filter to decide the correction.	N/A	N/A	C++
**Goldrush** ^**48**^	Graph-based (multi-purpose)	*de novo*	Custom polisher GoldPolish to correct low quality bases, Tigmint-long to correct misassemblies.	N/A	N/A	C++, Python3
**MetaFlye** ^**42**^	Graph-based (strain-aware)	*de novo*	Thresholding of low-frequency k-mers with Poisson error distribution model	Iteratively condense read graph	N/A	C++, Python
**Strainline** ^**28**^	Graph-based (strain-aware)	*de novo*	local de Bruijn graph-based strategy	Read clustering, iterative extension by OLC algorithm	Calculate depth of coverage of each haplotype based on alignment against input reads	Python
**HaploDMF** ^**43**^	Probabilistic	Reference-based	Post-process of assembled sequence with Medaka polisher.	Hierarchical clustering algorithm and Deep matrix factorization	based on the number of reads within each cluster	Python3
**iGDA** ^**44**^	Probabilistic	Reference-based	N/A	Estimate number of clusters with ANN algorithm and perform contigs extension using overlapping of SNVs.	Based on depth of coverage of each SNV site	C++
**RVHaplo** ^**45**^	Probabilistic	Reference-based	Post-process of assembled sequence with Medaka polisher.	Hierarchical clustering and generate consensus for each cluster	based on the number of reads within each cluster	Python3

Basic information of the assemblers is shown. N/A indicates no information provided by developer.

### Computational systems

Benchmarking analyses were conducted on three different computational systems: a server, a workstation PC, and a standard home PC. Specifications of each system is indicated in Table 2. The performance parameters of each assembler were measured as follows: 1) total CPU utilization, indicating the overall workload on the CPU, 2) memory usage, and 3) total runtime, defined as the elapsed wall-clock time from the start of assembly to the generation of output contigs. A dedicated Python script was developed to track the resource usage of each process of the long-read assemblers, capturing CPU and memory utilization every 100 milliseconds at each stage of the assembly process. As the pipeline operates within a containerized environment facilitated by Docker, unrelated processes such as operating system operations were filtered out during the measurement of resource utilization.

**Table 2.** Computational systems and specifications.

System	CPU	Memory	OS
Server	Intel(R) Xeon(R) Gold 6248 CPU @ 2.50 GHz	189G	Ubuntu 20.04.6 LTS
Workstation PC	Intel(R) Core i7-8700 CPU @ 3.20 GHz	DIMM DDR4 Synchronous 2133 MHz x4 (total 64 Gb)	Debian11
Generic Home PC	AMD RYZEN5 2600 3.4 GHz	8GB DDR4	Windows 10 on Docker with Linux Subsystem

### HIV-1 genome mixtures

Long-read FASTQ files were simulated from four HIV-1 genome mixtures: (1) 2 group M HIV-1 subtypes (2M), (2) 2 CRF subtypes (2C), (3) 1 group M HIV-1 subtype and 1 CRF subtype (1M1C), and (4) 2 group M HIV-1 subtypes and 1 CRF subtype (2M1C), see Table 3. Each genome mixture comprised 100 sets of FASTA files containing corresponding HIV-1 genomes randomly selected from the Los Alamos HIV sequence databases ^53^ ( https://www.hiv.lanl.gov/). All 400 HIV-1 FASTA sets underwent data simulation to generate 400 individual long-read FASTQ files using NanoSim V3.1.0. A list of mixtures within the 400 FASTA sets is available in Extended data, Table S6. ^54^ Additionally, 20 long-read FASTQ files, comprising 5 samples from each mixture ( Table 3), were generated with varying amplicon sizes as described in the assembly quality assessment.

**Table 3.** The HIV-1 genome mixtures and the simulated FASTQ files.

Experiment	Data	Detail	Number of FASTQ	Average read length (nt)	Number of reads per FASTQ
**Minimum read length**	**1,000-nt**	5 FASTQ files for each one of 4 HIV-1 mixtures, e.g., 2M, 2C, 1M1C, and 2M1C mixture.	20	1,120.715	2,000
	**2,000-nt**	5 FASTQ files for each one of 4 HIV-1 mixtures, e.g., 2M, 2C, 1M1C, and 2M1C mixture.	20	2,015.835	2,000
	**3,000-nt**	5 FASTQ files for each one of 4 HIV-1 mixtures, e.g., 2M, 2C, 1M1C, and 2M1C mixture.	20	3,022.300	2,000
	**4,000-nt**	5 FASTQ files for each one of 4 HIV-1 mixtures, e.g., 2M, 2C, 1M1C, and 2M1C mixture.	20	3,917.050	2,000
**HIV-1 mixture**	**2M**	2 Group M HIV-1 subtypes	100	8,316.558	2,000
	**2C**	2 HIV-1 CRFs	100	8,704.532	2,000
	**1M1C**	1 Group M HIV-1subtype and 1 CRF	100	8,366.96	2,000
	**2M1C**	2 Group M HIV-1 subtypes and 1 CRF	100	8,298.88	2,000

The characteristics of the simulated FASTQ files for the minimum read length dataset can be found in Extended data, Table S7. ^54^ The information about the HIV-1 genome mixtures is provided in Extended data, Table S8. ^54^

### Data simulation and experimental data

The data simulations were conducted using NanoSim V3.1.0 ( https://github.com/bcgsc/NanoSim/releases/tag/v3.1.0) in metagenomic mode, ^55^^,^^56^ employing a pretrained read profile model named human NA12878 DNA FAB49712 guppy. Additional simulation conditions included a maximum read length of 12,000 nt, a minimum read length of 7,500 nt, a median read length of 9,000 nt, and a standard deviation of read length set to 0.75. The parameter “--perfect” was configured as True to simulate error-free reads. A depth of coverage of 2,000 was selected, deemed sufficient for variant calling and quasispecies detection. ^57^^,^^58^ The resulting FASTQ files ( Table 3) were similar to those generated by an Oxford-ONT R9.4 chemistry using a Guppy 3.1.5 basecaller. Furthermore, this study benchmarked the assemblers using experimental data sourced from published studies (Extended data, Table S1). ^54^ All FASTA templates for data simulation, ^59^ simulated FASTQ files, ^60^ and QC results of the simulated FASTQ files ^61^ are available as Underlying data.

### Assembly quality evaluation

Sequence similarity was assessed using local NCBI BLASTN ^62^ against a customized database of 12,000 HIV-1 genomes from the Los Alamos HIV-1 sequence database, ^53^ with contigs showing >95% sequence similarity considered. QUAST ^63^ was utilized to compare contigs from different assemblers, with regular QUAST for single viral isolate datasets and MetaQUAST ^63^ for multiple isolate inputs. Primary QUAST parameters assessed were number of contigs, sizes, N50, % genome fraction, and total aligned bases, along with assembly correctness measured by average mismatches and indels per 100,000 aligned bases. Additionally, assembly quality was evaluated by average completeness of major HIV-1 ORFs (e.g., *gag*, *gag-pol/pol*, and *env*). The results generated from the pipeline are available as Underlying data. ^64^

## Results

### Evaluating the computational performance of the assemblers

**Figure 2.**
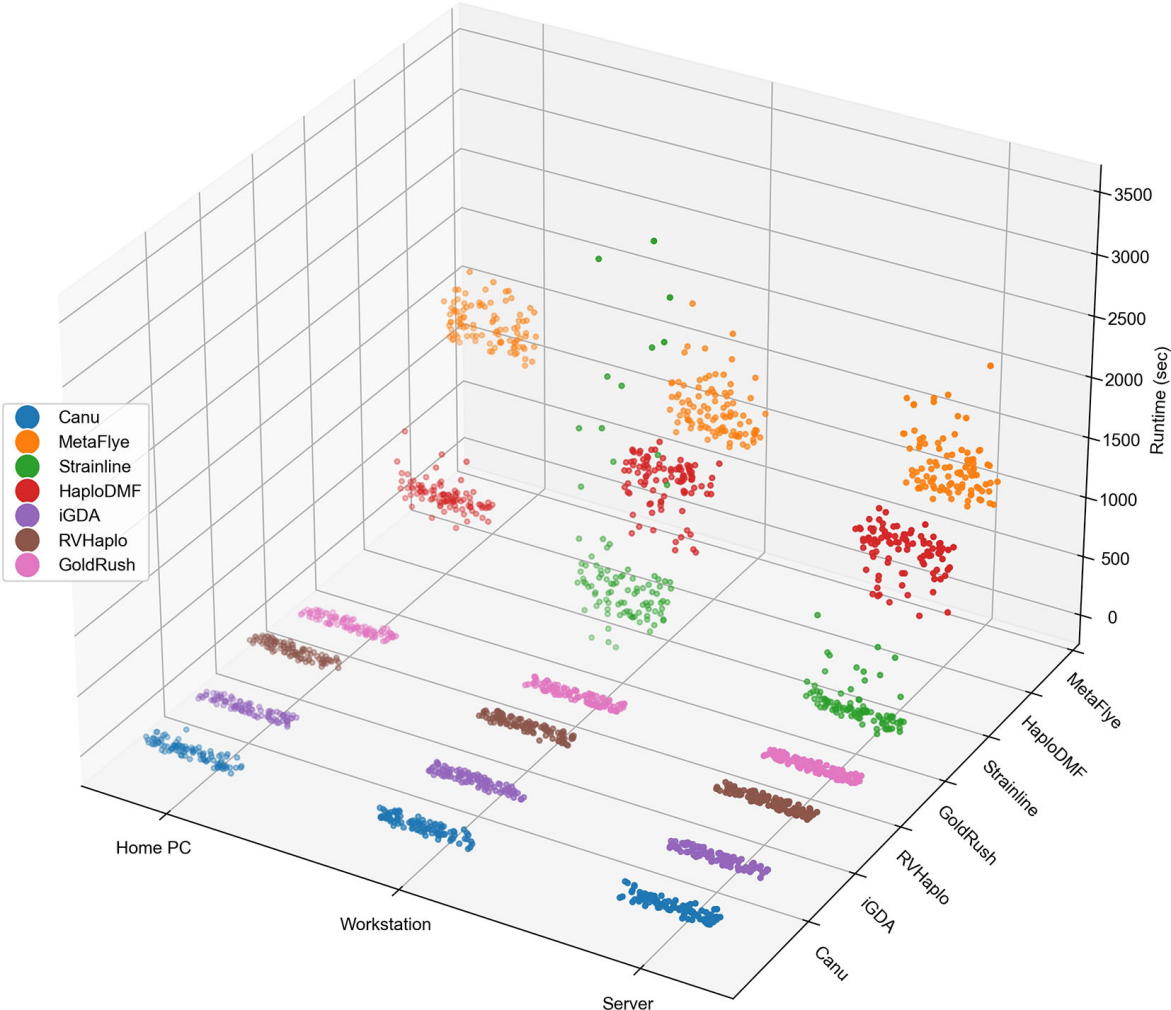
A depiction of time usage of all 2,100 individual assemblies. The assemblies (100 simulated 2M FASTQ files per assembler per system) are grouped by computational systems on the x-axis and by assemblers on the y-axis. Additionally, an accompanying statistical summary is provided in Extended data, Table S2. ^54^

All seven assemblers were executed on three computational systems to measure wall-clock time usage, memory usage, and CPU utilization. Each assembler processed 100 simulated 2M FASTQ files. Figure 2 illustrates the time usage of all FASTQ files processed by different assembly pipelines across the three computational systems. As shown in Extended data, Table S2, ^54^ the workstation server completed all 700 assemblies in 3 days, 9 hours, 50 minutes, and 28.31 seconds, while the workstation PC required 3 days, 23 hours, 24 minutes, and 2.64 seconds. Due to a failed Strainline-mediated assembly with the generic home PC, the remaining 600 assemblies took 2 days, 14 hours, 49 minutes, and 16.79 seconds. Notably, GoldRush emerged as the fastest assembler, with approximately 30 seconds or less per assembly under all three computational systems, while MetaFlye was the slowest, taking approximately 1,370 seconds or more per assembly.

**Figure 3.**
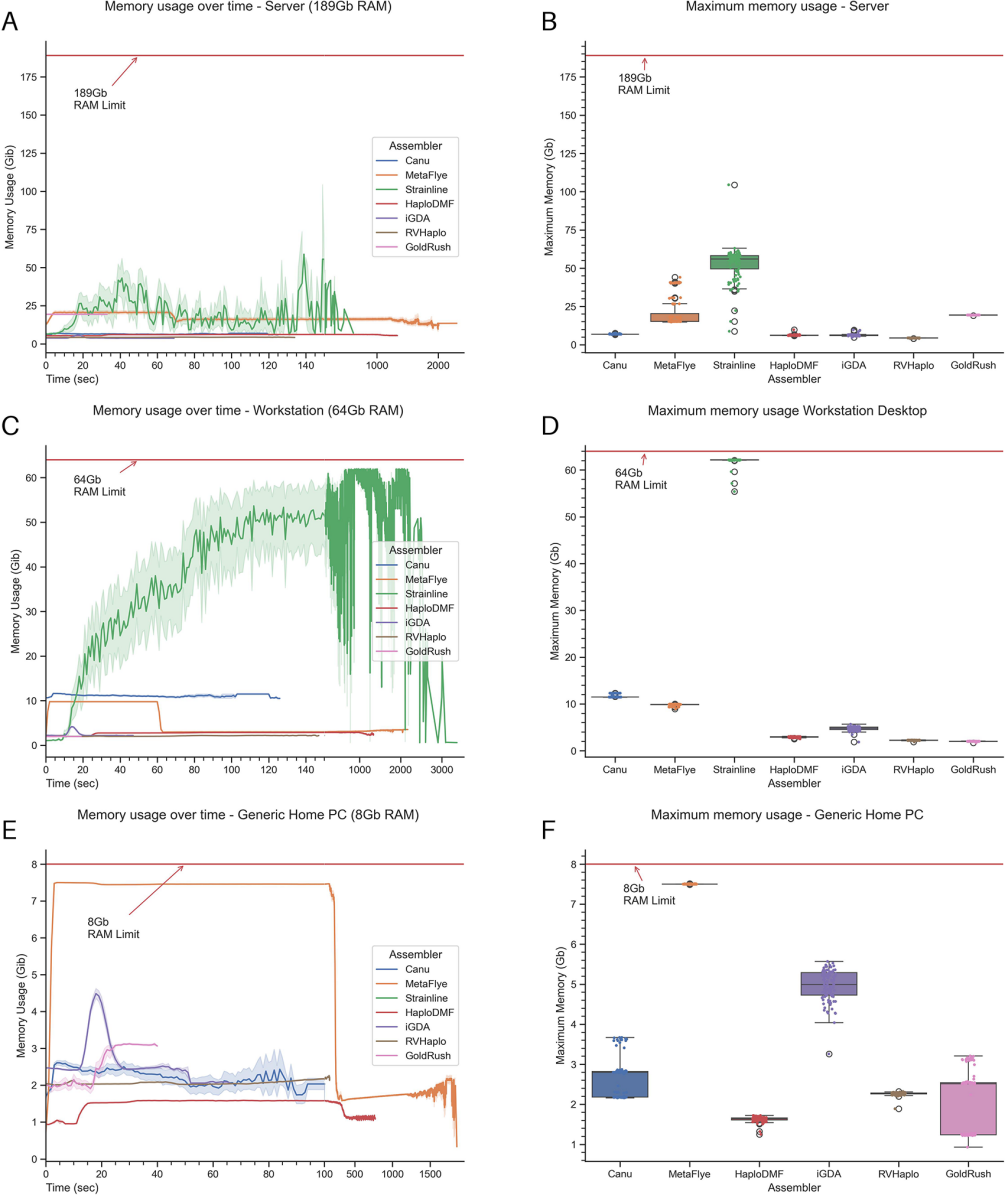
Memory usage overtime and maximum memory usage. Memory usages of all assemblers under three computational systems, including (A) and (B) a workstation server, (C) and (D) a workstation PC, and (E) and (F) a generic home PC during the assembly phase of the pipeline.

For memory usage, Strainline could not be tested with the generic home PC due to a segmentation fault error from memory limitation. From the 189-Gb workstation server, the order of maximum memory usage, from highest to lowest, was Strainline, MetaFlye, GoldRush, HaploDMF, iGDA, Canu, and RVHaplo ( Figure 3A and B). Similarly, from the 64-Gb workstation PC, the order of maximum memory usage was Strainline, Canu, MetaFlye, HaploDMF, iGDA, RVHaplo, and GoldRush ( Figure 3C and D). From the 8-Gb generic home PC, the order of maximum memory usage was MetaFlye, iGDA, Canu, GoldRush, RVHaplo, and HaploDMF ( Figure 3E and F). Extended data, Table S3 ^54^ presents the maximum memory usages of the six assemblers.

**Figure 4.**
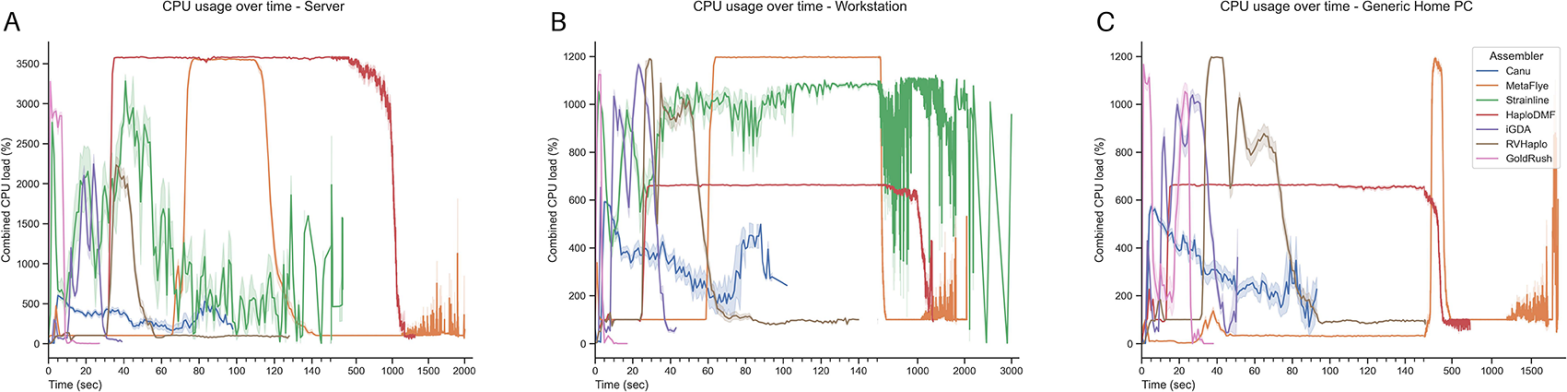
CPU usages of all seven assemblers under three different computational systems. In this study, the server could accommodate 36 cores (3,600%), while both the workstation PC and the generic home PC could accommodate up to 12 cores (or 1,200%). CPU usages were collected from (A) a server, (B) a workstation PC, and (C) a generic home PC. The y-axis represents percent CPU usage (100% per core). Multi-threaded processing can be observed when CPU utilization is above 100%.

Figure 4 demonstrates the CPU utilizations of all assemblers, with the percentages of maximum CPU usages shown in Extended data, Table S3. ^54^ On the workstation server ( Figure 4A), the assemblers with the highest to lowest averaged CPU usage were HaploDMF, Strainline, GoldRush, iGDA, RVHaplo, Canu, and MetaFlye. Both GoldRush (3,625.9%) and Canu (647.4%) did not overutilize the CPU and remained within the acceptable limit for 36 cores. On the workstation PC ( Figure 4B), the order of CPU usage was Strainline, HaploDMF, iGDA, Canu, RVHaplo, GoldRush, and MetaFlye. Notably, MetaFlye overutilized CPU at 1,437.4%, whereas Canu demonstrated only 637.5% peak utilization. Under the generic home PC system ( Figure 4C), the CPU usage order was GoldRush, iGDA, RVHaplo, MetaFlye, Canu, and HaploDMF. Both HaploDMF (726.3%) and Canu (628.9%) did not overutilize the CPU. Finally, Correlations between computational factors (e.g., CPUs, maximum memory, and assemblers) and runtime were analyzed using Jamovi statistical software with a non-parametric one-way ANOVA. ^65^ *^,^* ^66^ As demonstrated in Table 4, assembler selection significantly impacted runtime with a large effect size (ε ^2^ = 0.903***), while no statistically significant differences in runtime were observed across different levels of CPUs and maximum memory.

**Table 4.** A statistical correlation analysis between computational factors and runtime.

Correlation	χ ^2^	df	p	ε ^2^
CPUs – Runtime	0.217	1	0.641	1.09e-4
Memory – Runtime	0.921	2	0.631	4.61e-4
Assembler – Runtime	1,805	6	< 0.001***	0.903

Kruskal-Wallis (***p-value <0.001).

### Determining the minimum read lengths required for the assemblers

The minimum read length was determined by testing the assemblers with FASTQ files from four different median read lengths: 1,000-nt, 2,000-nt, 3,000-nt, and 4,000-nt ( Table 3). This assessment aimed to ensure that the assembled contigs exhibited high contiguity, genome completeness, and recovered intact major HIV-1 open reading frames (ORFs) such as gag, gag-pol/pol, and env. The evaluation was conducted using the server system.

**Figure 5.**
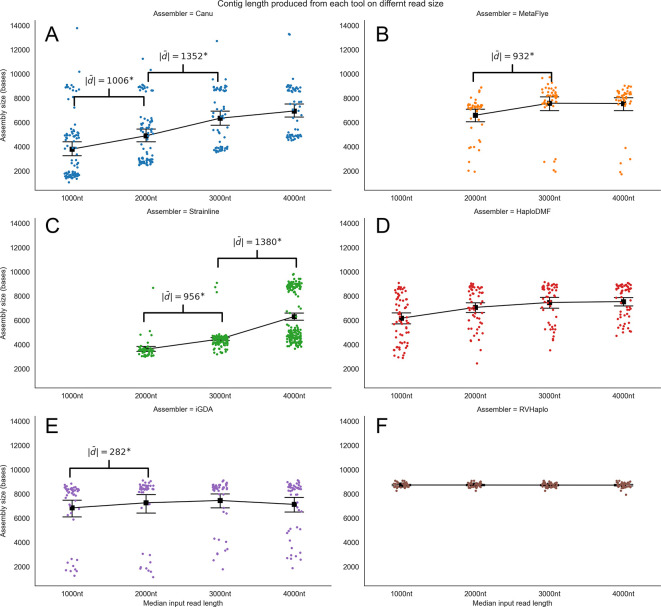
Contig size distribution from the assemblers processing four median read length FASTQ inputs. The pipelines were (A) Canu, (B) MetaFlye, (C) Strainline, (D) HaploDMF, (E) iGDA, and (F) RVHaplo. The x-axis represents four median read lengths (1,000–4,000 nt), and the y-axis represents contig size. Each data point denotes an individual contig. The mean contig size of each median read length is indicated by a black square with error bars representing 95% confidence intervals. Absolute mean differences are shown in brackets for those comparisons with statistical significance (p<0.001).

The first evaluation of the minimum read lengths was based on the assembled contig size. Increasing median read lengths resulted in longer contigs assembled by Canu ( Figure 5A). MetaFlye ( Figure 5B) and Strainline ( Figure 5C) could not process the 1,000-nt inputs, but Strainline assembled significantly longer contigs from longer reads. However, GoldRush failed the assessment. The remaining *de novo* assemblers, Canu, MetaFlye, and Strainline, processed the 4,000-nt median read length inputs and yielded contig sizes relatively close to a 9-kb HIV-1 genome. All reference-based assemblers performed well with all four inputs, generating contig sizes close to that of an HIV-1 genome ( Figure 5D-F). Complete statistical evaluation details can be found in Extended data, Table S9. ^54^

Sequence similarity and genome fraction of assembled contigs were evaluated across different median read lengths. For the 1,000-nt reads (Extended data, Figure S1A), ^54^ Canu yielded >5,000-nt contigs with >99.5% similarity, while iGDA and RVHaplo generated >8,000-nt contigs with 97% and 99% similarity, respectively. RVHaplo showed superior recovery of major HIV-1 ORFs (Extended data, Figure S2A). ^54^ At the 2,000-nt reads (Extended data, Figure S1B), ^54^ Canu and MetaFlye produced >7,000-nt contigs with >99% similarity, whereas Strainline’s contigs were ~3,000-nt with >98% similarity. All reference-based assemblers yielded longer contigs with greater similarity and improved ORF recovery (Extended data, Figure S2B). ^54^ At the 3,000-nt reads (Extended data, Figure S1C), ^54^ Strainline only produced 4,000-nt contigs with >99.50% similarity, while others generated longer contigs with higher similarity and improved ORF recovery (Extended data, Figure S2C). ^54^ Finally, at the 4,000-nt reads (Extended data, Figure S1D), ^54^ all assemblers produced longer contigs with higher similarity. All, except Canu and HaploDMF, demonstrated satisfactory % recovery of the major HIV-1 ORFs (Extended data, Figure S2D). ^54^ RVHaplo yielded the highest averaged genome fraction (Extended data, Figure S1E). ^54^

A further analysis ( Table 5) revealed a statistically significant positive correlation between the median read lengths of the inputs and the lengths of the assembled contigs (ρ=0.185***), as well as between the median read lengths and sequence similarity (ρ=0.163***). Additionally, assembler selection demonstrated a positive correlation with the size of assembled contigs (ρ=0.131***), but not with sequence similarity (ρ=-0.220). Thus, a key finding from this study suggested that a minimum read length of 2,000-nt is necessary for all assemblers, with optimal outcomes achieved at 4,000-nt. In summary, longer median read sizes are associated with higher quality contigs, characterized by longer contig length and greater sequence similarity.

**Table 5.** A statistical correlation analysis of the read lengths of inputs and outputs and sequence similarity.

		Median read length	Sequence similarity (%identity)	Assembler selection
Sequence similarity (%identity)	Spearman's rho	0.163 ***	—	
	df	1,243	—	
	p-value	< 0.001	—	
Assembler Selection	Spearman's rho	—	-0.220	—
	df	—	1,243	—
	p-value	—	1.000	—
Output contig length	Spearman's rho	0.185 ***	-0.290	0.131 ***
	df	1,243	1,243	1,243
	p-value	< 0.001	1.000	< 0.001

H _a_ is positive correlation

* p<0.05,

** p<0.01,

*** p<0.001, one-tailed.

### Evaluating the performance of assemblers using the four HIV-1 genome mixtures

This experiment evaluated the performance of assemblers in generating contigs from simulated FASTQ inputs containing heterogeneous HIV-1 sequences. These sequences were simulated from four HIV-1 genome mixtures: 2M (2 group M subtypes), 2C (2 circulating recombinant forms, CRFs), 1M1C (1 group M subtype and 1 CRF), and 2M1C (2 group M subtypes and 1 CRF), as shown in Table 3. The evaluation criteria included contiguity, genome completeness, the recovery of major HIV-1 open reading frames (ORFs), and the error rate. Initially, contig sizes were assessed, revealing that most fell within a range of 8,500–9,200 nt, close to the size of the HIV-1 genome (Extended data, Figure S3). ^54^ Notably, MetaFlye produced shorter contigs, ranging from 7,800 to 8,800 nt. Detailed statistical analysis of this assessment is available in Extended data, Table S10. ^54^

**Figure 6.**
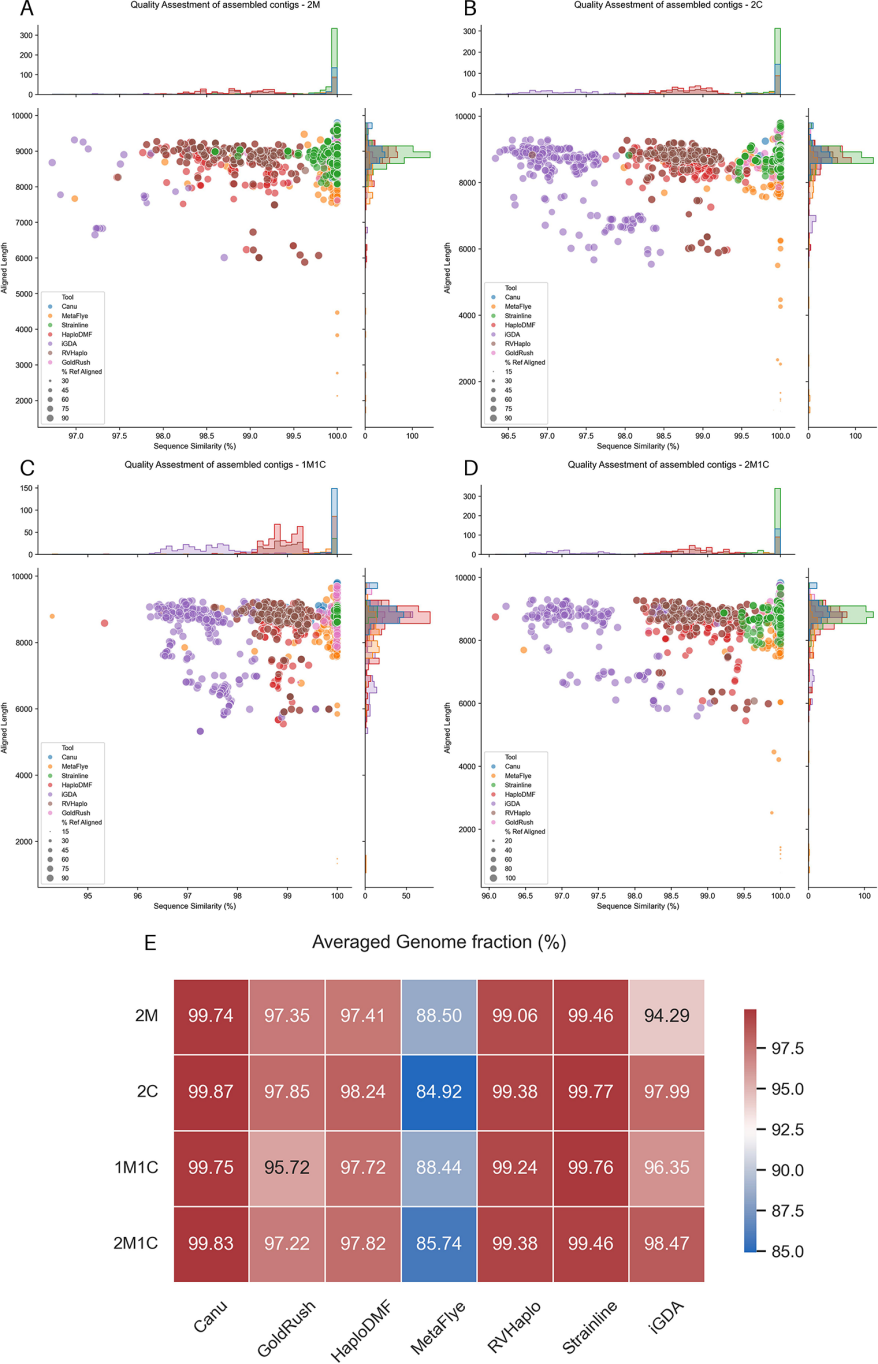
The MetaQUAST assessment evaluated contigs from the assemblers processing four sets of the simulated FASTQ. The FASTQ file sets were (A) 2M HIV-1 mixture, (B) 2C HIV-1, (C) 1M1C HIV-1 mixture, and (D) 2M1C HIV-1 mixture ( Table 3). The x-axis displays the percentage sequence similarity obtained from a BLAST alignment with the corresponding reference genomes, while the y-axis represents the aligned length, indicating the longest continuous alignment between each contig and its reference genome. Dot sizes indicate the genome fraction (%Ref Aligned), calculated as the ratio of continuous aligned bases to the total reference genome length. Subplots above and beside each figure display histograms of the contig counts. Additionally, (E) shows the averaged genome fraction (%) of contigs from different long-read assembler pipelines.

**Figure 7.**
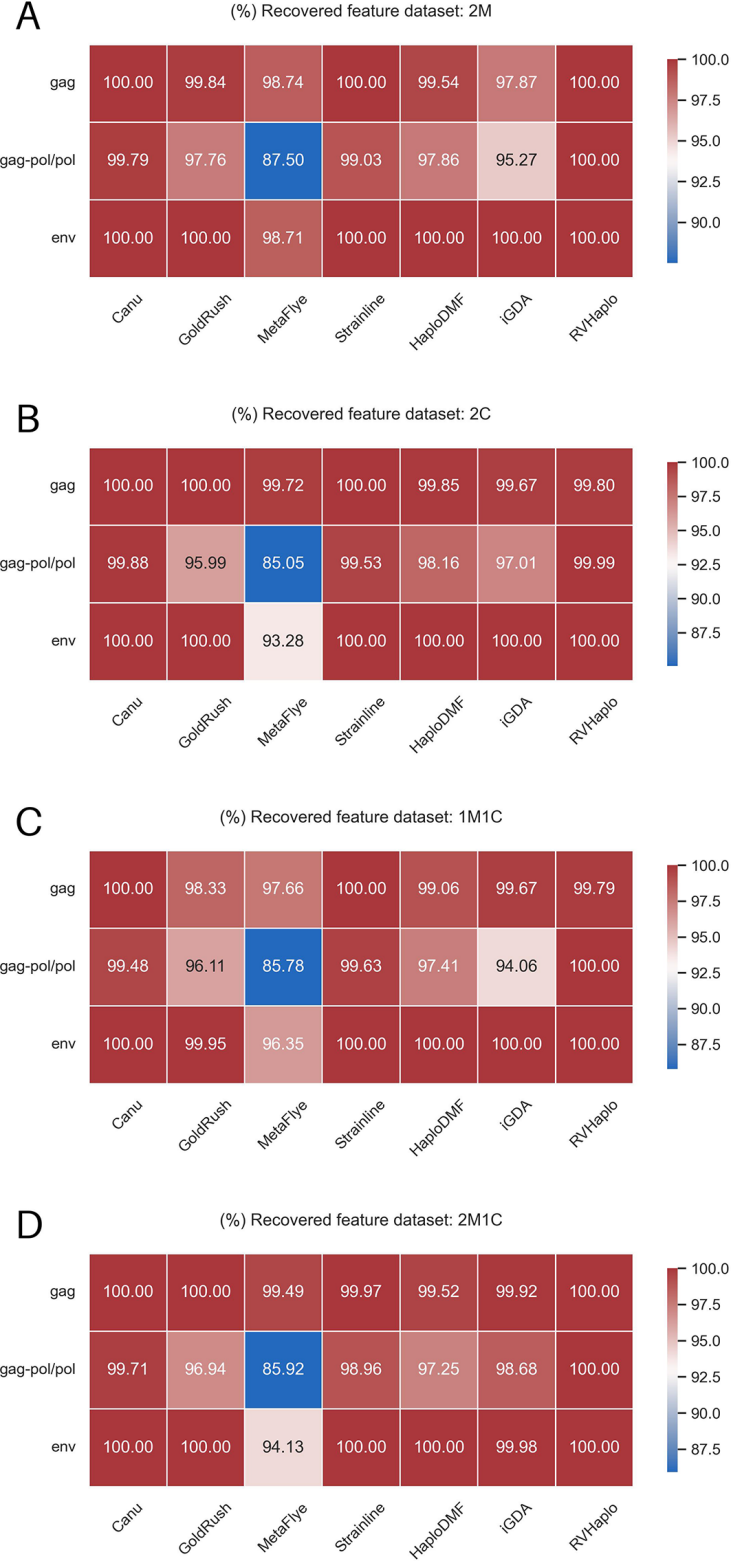
Averaged completeness of HIV-1 open reading frames (ORFs) of the contigs generated from the assemblers. The contigs were from the assemblers analyzed simulated FASTQ inputs of the 4 HIV-1 mixtures, e.g., (A) 2M, (B) 2C, (C) 1M1C, and (D) 2M1C.

Considering both contig length and averaged genome fraction, all assembler pipelines processed the four datasets to yield contigs exceeding 8,000 nt, close to the size of the HIV-1 genome ( Figure 6A–D). However, the MetaFlye pipeline generated contigs with a relatively low (<90%) averaged genome fraction ( Figure 6E). Except for MetaFlye, all assembler pipelines demonstrated satisfactory recovery of major HIV-1 ORFs ( Figure 7A–D). *De novo* and reference-based assemblers showed a clear distinction in 2M, 2C, and 2M1C HIV-1 mixtures ( Figure 6A, B, and D), with *de novo* assemblers producing contigs with >95.5% sequence similarity. Reference-based assemblers HaploDMF and RVHaplo yielded contigs with relatively high sequence similarity (98–99.5%), while iGDA produced 95–97% sequence similarity. However, iGDA generated fewer contigs from the 2M mixture inputs ( Figure 6A) due to its reliance on overlapping SNVs for contig extension, which may fail with highly similar sequences. The presence of CRFs allowed iGDA to recover more contigs ( Figure 6B–D). Individual assembly evaluation statistics for each sample are available in Extended data, Table S10. ^54^

**Figure 8.**
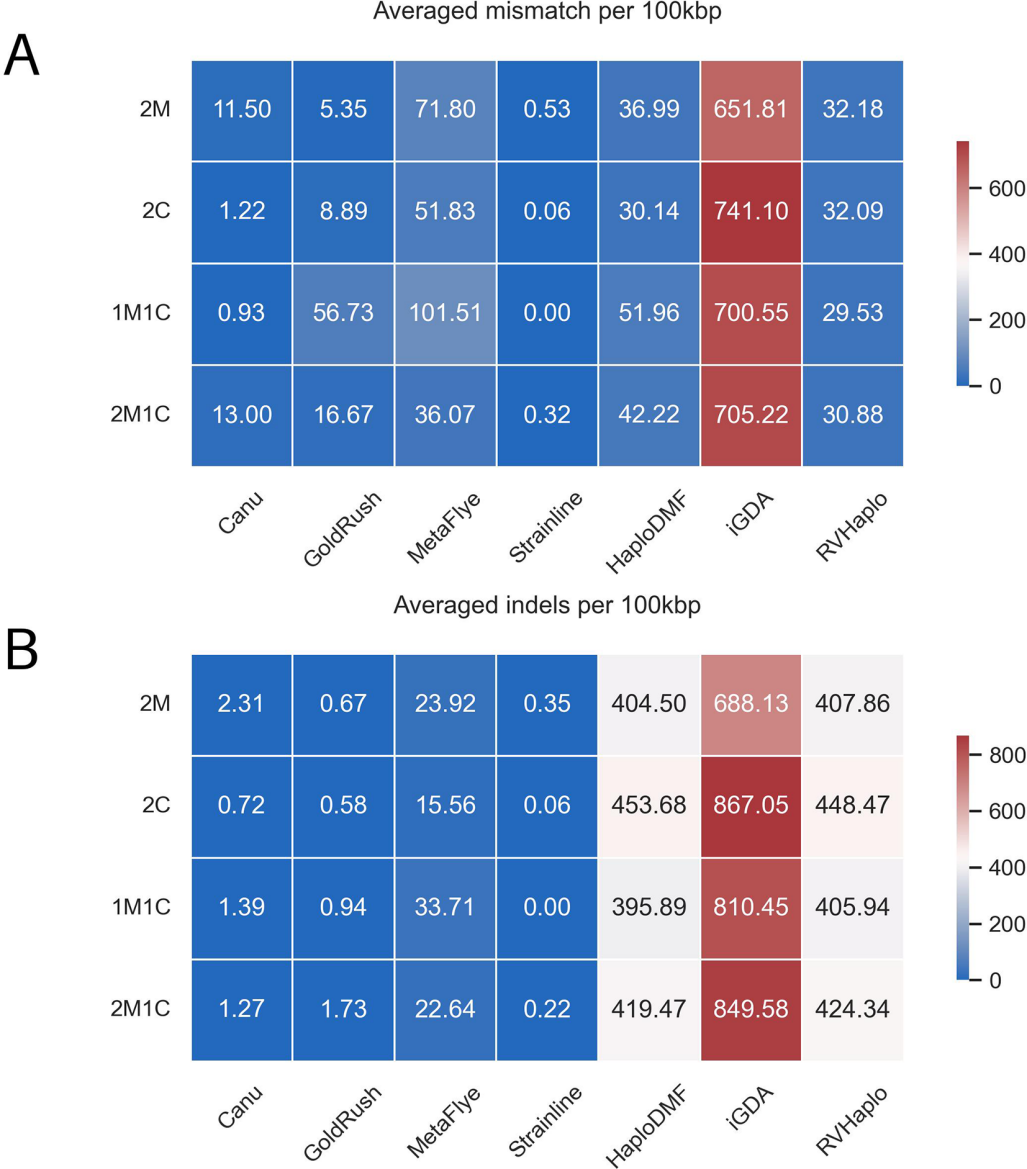
The errors observed in the contigs from the simulated FASTQs of the 4 HIV-1 mixtures. The errors were (A) the average number of mismatches (e.g., true SNPs and sequencing errors) per 100,000 aligned bases and (B) the average number of indels per 100,000 aligned bases.

Since all input data consisted of error-free reads, the assembly correctness ( Figure 8) was assessed by comparing the contigs with their respective HIV-1 genomes. The correctness of each assembler was evaluated based on the average number of mismatches per 100,000 aligned bases ( Figure 8A) and the average number of indels per 100,000 aligned bases ( Figure 8B). Among the four *de novo* assemblers, all introduced a few errors to the contigs, with Strainline exhibiting a greater degree of accuracy compared to the rest. MetaFlye, however, showed the least assembly correctness. Regarding the reference-based assemblers, HaploDMF and RVHaplo demonstrated similar degrees of assembly correctness, while iGDA exhibited the least. Interestingly, indels were predominantly associated with all reference-based assemblers in this study.

**Figure 9.**
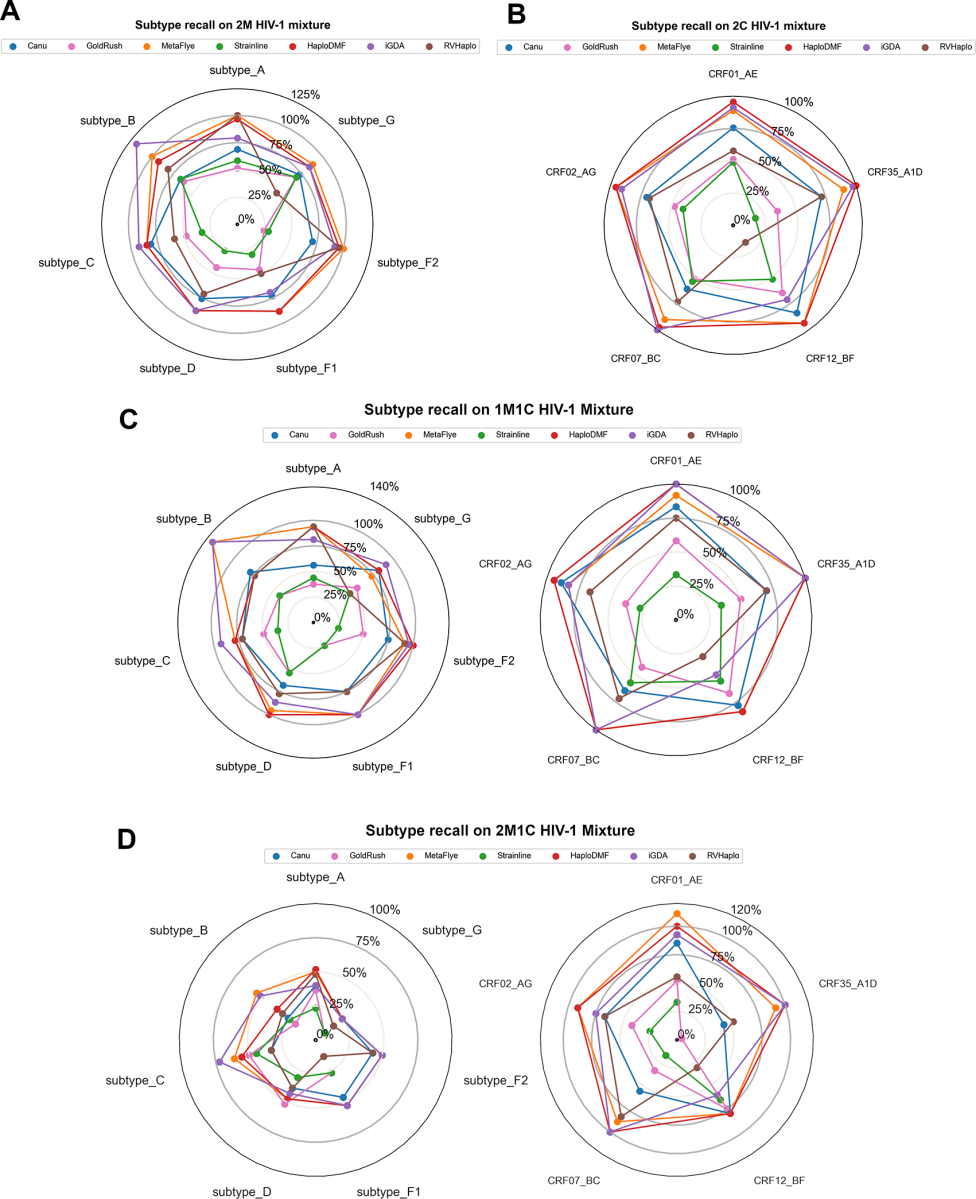
HIV-1 subtypes and CRFs recall rate by each assembler. Subtype recall rate of each subtype or CRF in (A) 2M HIV-1 mixture, (B) 2C HIV-1 mixture, (C) 1M1C HIV-1 mixture, and (D) 2M1C HIV-1 mixture. Each assembler's recall rate was calculated from the number of correctly identified HIV-1 subtype (or CRF) divided by a total number of a corresponding subtype (or CRF) appearing in each HIV-1 mixture. The value of over 100% was from extra contigs of that subtype produced from mixing closely related subtypes together. See Extended data, Table S11 ^54^ for more details.

The final assessment investigated the HIV-1 subtype recall rates of the assemblers processing the 4 HIV-1 mixture datasets. In the 2M dataset ( Figure 9A), the reference-based assemblers, such as HaploDMF, RVHaplo, and iGDA, correctly recalled most of the subtypes, while the *de novo* assemblers exhibited lower recall rates. Strainline showed the best performance with >80% recall rates on all common subtypes, except for an overestimation on subtype B (118.52%). Canu, GoldRush, and MetaFlye exhibited low recall rates due to their collapsed assembly approach poorly handling sequences of relatively similar HIV-1 subtypes. Results from the 2C ( Figure 9B) and the 1M1C data ( Figure 9C) were similar to those observed in the 2M data. Interestingly, results from the 2M1C data ( Figure 9D) showed much lower recall rates (<60%) by all assemblers on all HIV-1 group M subtypes due to either a reduction in coverage or an error correction process. For the CRFs, the recall rates were similar to those shown in the 2C and 1M1C datasets.

Based on the results from the previous assessments, Strainline, MetaFlye, and HaploDMF were selected for further investigation using publicly available experimental sequencing data of HIV-1 and other viruses. Strainline, despite its high memory usage, offered rapid *de novo* viral haplotype reconstruction. MetaFlye, chosen for its compatibility with lower-end devices and acceptable memory usage and runtime, served as a *de novo* assembler. Despite its long runtime, HaploDMF was included as a representative reference-based assembler with memory efficiency comparable to MetaFlye.

### Assessing the performance of three selected long-read assembler pipelines using experimental data from long-read sequencing of HIV-1 and other viruses

This experiment assessed the performance of selected assemblers, including Strainline, MetaFlye, and HaploDMF, using experimental data of HIV-1 and other pathogenic viruses (Extended data, Table S1). ^54^ The quality of assembled contigs was evaluated through QUAST, which provided metrics such as the number of contigs, sizes, N50, % genome fraction, and total aligned bases. The experiment was conducted using a workstation server.

*HIV-1 recombinant forms and NL4-3*

**Figure 10.**
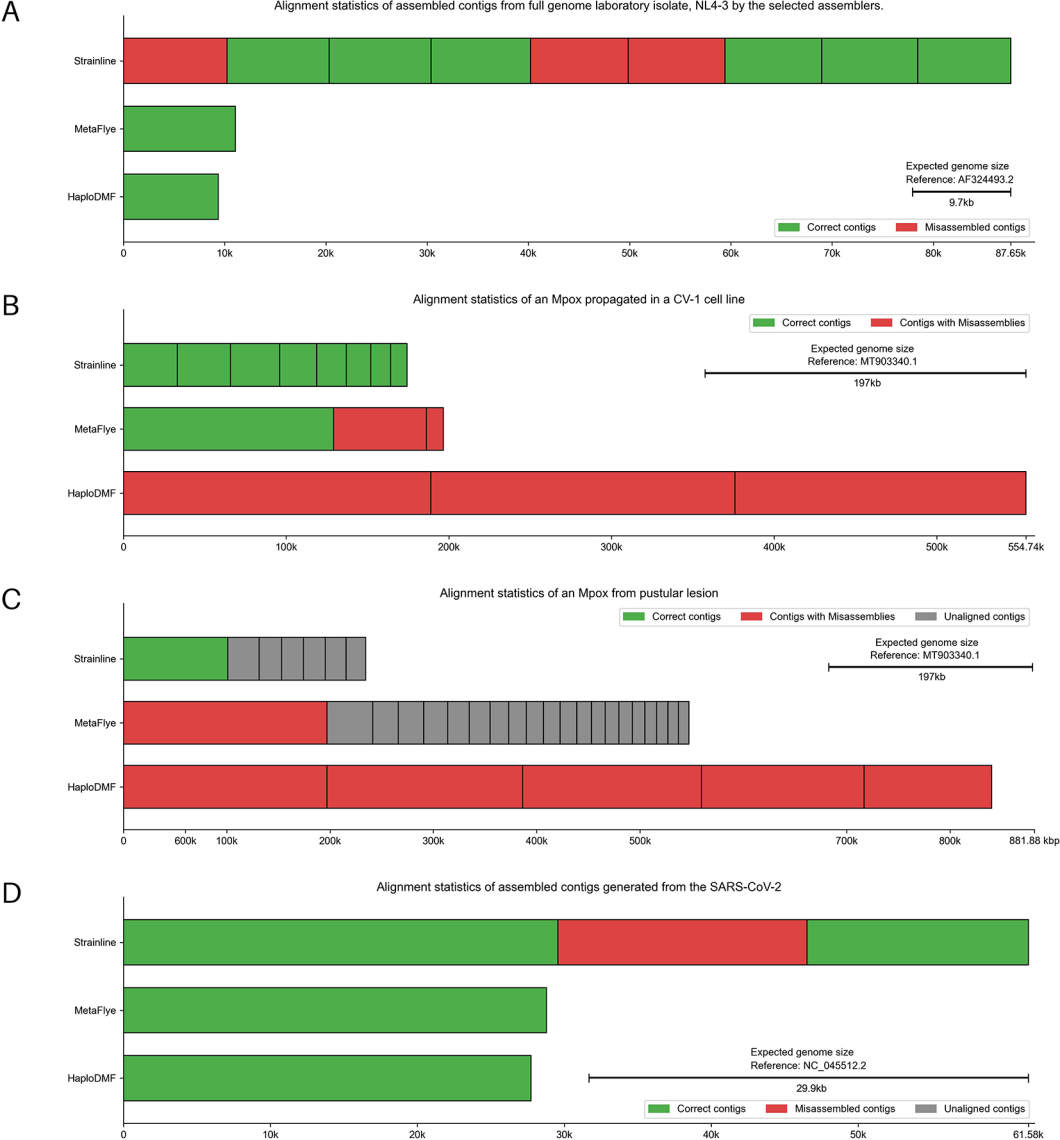
Alignment statistics of assembled contigs generated from HIV-1, SARS-CoV-2, and Mpox sequencing data. The assembled contigs were from (A) Full genome laboratory isolate NL4-3, (B) Mpox propagated in a CV-1 cell line and (C) Mpox from pustular lesion, and (D) SARS-CoV-2 sequencing data by the selected assemblers. The details of samples and reference genomes are shown in Extended data, Table S1. ^54^ Visualization modified from Icarus. ^63^ Individual alignment statistics for each haplotype can be found in Extended data, Table S14. ^54^

The dataset comprised plasma HIV-1 genomes (TRN01, TRN08, TRN09) and NL4-3 laboratory isolate from the MinION sequencer (BioProject: PRJDB13369). ^67^ All assemblers generated NL4-3 contigs >9,000 nt ( Figure 10A). MetaFlye and HaploDMF gave contigs with 95.27% and 91.22% sequence similarity, respectively. Strainline produced nine contigs, the best at 94.34% similarity (Extended data, Table S4). ^54^ For TRN01, all assemblers produced contigs identified by REGA (version 3.46) ^68^ as subtype B and F recombinants, similar to a previous finding. ^67^ Similarly, TRN08 yielded subtype B contigs. However, no recombinant forms with CRF01_AE were reconstructed. For TRN09, Strainline and HaploDMF replicated original findings, while HaploDMF also identified an A-C recombinant. In contrast, MetaFlye generated subtype A and CRF07_BC gagpol contigs, and a C-CRF01_AE recombinant. REGA subtyping results are in Extended data, Table S12. ^54^ For the 10 5’ half genomes of NL4-3 (BioProject: PRJNA938445), ^69^ all assemblers produced contigs meeting both length and alignment criteria. MetaFlye achieved the highest performance with an 89.26% average genome fraction, followed by Strainline at 85.93% and HaploDMF at 84.64%. Detailed alignment statistics are provided in Extended data, Table S13. ^54^

*Monkeypox (Mpox)*

The first sequencing data, derived from MinION sequencing of a Mpox propagated in a CV-1 cell line (SRA: ERR10963128), ^70^ contained 2,588 reads post-filtering, with an average length of 4,633 nt and a maximum length of 9,070 nt. HaploDMF generated 3 contigs, the longest being 188,872 nt, with 99.35% genome fraction. MetaFlye produced 7 contigs, the longest at 129,209 nt, with 95.98% genome fraction. Strainline yielded 12 contigs, the longest being 33,081 nt, with a genome fraction of 78.07% ( Figure 10B and Extended data, Table S4 ^54^). The second dataset, derived from MinION sequencing of a Mpox from a pustular lesion (SRA: SRR15830920), ^71^ comprised 30,990 reads after filtering, with an average length of 9,048 nt and a maximum length of 59,093 nt. MetaFlye and HaploDMF exhibited similar performance, with MetaFlye assembling 122 contigs with an N50 of 13,335 nt and the longest having a 98.58% genome fraction. HaploDMF produced 5 contigs with an N50 of 173,134 nt and the longest contig having a genome fraction of 98.62%. In contrast, Strainline generated 7 contigs with an N50 of 30,255 nt and the longest contig of 101,047 nt. Only the 65,261-nt contig of those 7 contigs demonstrated 51.31% genome fraction ( Figure 10C and Extended data, Table S4 ^54^).

*Severe Acute Respiratory Syndrome Coronavirus 2 (SARS-CoV-2)*

The dataset comprises GridION sequencing data of a SARS-CoV-2 clinical sample retrieved from the SRA database (SRP250446). ^72^ Strainline generated 3 contigs: 1 full-length and 2 duplicated half-genome contigs, with a 99.93% genome fraction. MetaFlye and HaploDMF each produced a single contig, with genome fractions of 96.77% and 93.11%, respectively. However, MetaFlye’s contig lacked the first 500 nucleotides of the genome, while HaploDMF’s contig had a 2,000-nt gap when aligned to the reference genome ( Figure 10D and Extended data, Table S4 ^54^).

*Polio virus mixture*

**Figure 11.**
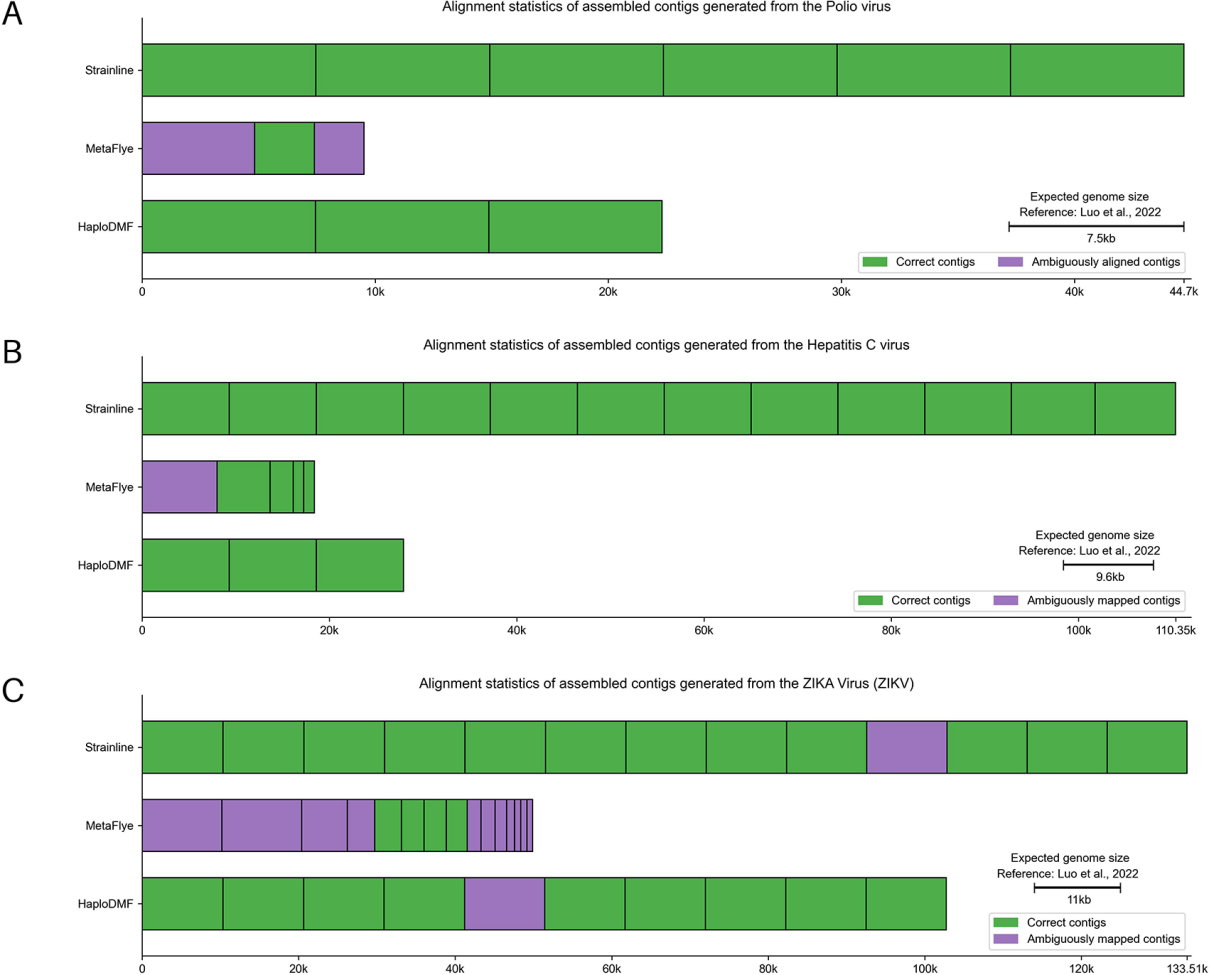
Alignment statistics of assembled contigs generated from simulated mix-strain viral sequencing data. The assembled contigs were from simulated mix-strain viral sequencing data of (A) Polio virus, (B) Hepatitis C virus (HCV), and (C) ZIKA Virus (ZIKV) by the selected assemblers. The details of samples and reference genomes are shown in Extended data, Table S1. ^54^ Visualization modified from Icarus. ^63^ Individual alignment statistics for each haplotype can be found in Extended data, Table S14. ^54^

This simulated ONT dataset consisted of 6 poliovirus type 2 sequences. ^28^ In Figure 11A and Extended data, Table S5, ^54^ Strainline and HaploDMF generated contigs with N50 values of 7,453-nt and 7,436-nt, respectively, closely matching the 7,400-nt genome of Polio virus. Strainline produced 6 contigs with an average genome fraction of 99.34%, all sharing >95% sequence similarity with 6 viral haplotypes. HaploDMF yielded 3 contigs with >95% sequence similarity and an average genome fraction of 99.80%. MetaFlye, however, produced 3 shorter contigs with an N50 of 4,830-nt. These contigs shared >95% sequence similarity with 5 haplotypes, but with an average genome fraction of 70.13%.

*Hepatitis C virus (HCV) mixture*

This simulated ONT dataset consisted of 10 strains of hepatitis C virus (HCV), Subtype 1a. ^28^ In Figure 11B and Extended data, Table S5, ^54^ Strainline and HaploDMF yielded contigs with N50 values of 9,286-nt and 9,309-nt, respectively. Strainline produced 12 contigs with an average genome fraction of 99.85%, all of them sharing >95% sequence similarity with all 10 haplotypes with 2 duplicated contigs. HaploDMF generated 3 contigs with a 99.99% average genome fraction, all exhibiting >95% sequence similarity with HCV haplotypes. MetaFlye produced 5 shorter contigs with an N50 of 5,670-nt, and the longest contig (8,006-nt) shared >95% sequence similarity with 5 HCV haplotypes. However, MetaFlye contigs had an average genome fraction of 77.40%.

*ZIKA Virus (ZIKV) mixture*

This simulated ONT dataset comprised 15 strains of Zika virus (ZIKV). ^28^ In Figure 11C and Extended data, Table S5, ^54^ Strainline, MetaFlye, and HaploDMF generated contigs with N50 values of 10,264-nt, 5,842-nt, and 10,264-nt, respectively, closely matching the size of the ZIKV genome (11,000-nt). Strainline produced 13 contigs that recovered 14 ZIKV haplotypes, with an average genome fraction of 99.84%. MetaFlye yielded 15 contigs with a lower average genome fraction of 78.56%, recovering 10 ZIKV haplotypes. Interestingly, HaploDMF produced 10 contigs, each with an average genome fraction of 99.90%, and recovered 11 ZIKV haplotypes.

In summary, the selected assemblers showed strong performance across different experimental ONT datasets. MetaFlye exhibited superior performance with experimental ONT data of full-length or half-length HIV-1 genomes. Strainline demonstrated excellent haplotype recovery with ONT sequencing data of various viral mixtures, including SARS-CoV-2, Poliovirus, HCV, and ZIKV. Additionally, MetaFlye showcased better performance on the metagenomic experimental ONT data of the 200-kb Mpox.

### A containerized HIV-64148 pipeline for genomic surveillance

**Figure 12.**
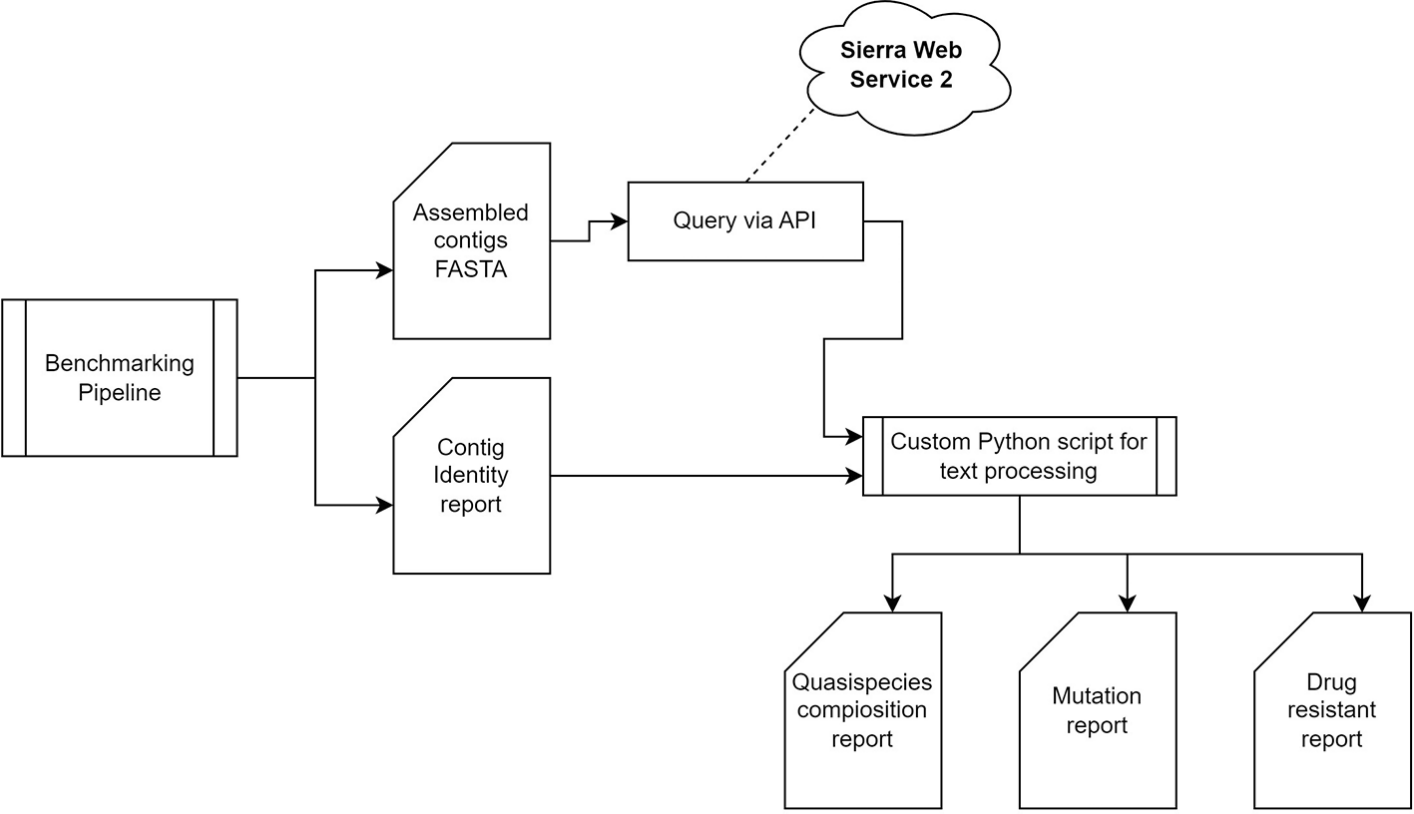
An implementation of the HIV-64148 pipeline for genomic surveillance. The pipeline comprises three main stages: a read quality control analysis of long-read FASTQ files, followed by assembly using either *de novo* or reference-based assemblers, and concluding with the identification of HIV-1 subtype and drug resistance analysis.

The HIV-64148 benchmarking pipeline is designed for portability, ensuring it functions across different computational environments with minimal setup. As a result, the containerized HIV-64148 can be executed on either Docker or Singularity platforms. The pipeline offers the flexibility to select from a range of assemblers—Canu, GoldRush, MetaFlye, Strainline, HaploDMF, iGDA, and RVHaplo—to best match the computational system or meet specific study requirements. It generates a comprehensive report in HTML format, detailing contigs/haplotype identities, and abundances. Additionally, the report includes protein sequence alignments of drug-targeted HIV-1 genes, alongside profiles of drug resistance and susceptibility associated with these genes. This is achieved by querying the assembled sequence against the Stanford University HIV Drug Resistance Database ^73^ *^–^* ^78^ via the provided Sierra web service 2 API, as illustrated in Figure 12. The downstream analysis pipeline could also serve as a model for implementation with other viruses. The source code for building the containerized pipeline is available in a public code repository: https://github.com/STTLab/HIV-64148.

## Discussion

In this study, we benchmarked seven selected long-read assemblers using both simulated and experimental long-read sequencing data of HIV-1 and other viruses on three computational systems to assess their performances. We demonstrated that only the assembler selection exerts a statistically significant impact on assembly time, with neither CPU nor memory affecting the process. ^79^ Assembler selection also influences the size of assembled contigs. Additionally, a minimum read length is 2,000-nt, and the 4,000-nt read length results in higher quality assembled contigs. Further investigations revealed that Strainline, MetaFlye, and HaploDMF deliver successful genome assemblies of long-read sequencing data, exhibiting sequence heterogeneity, from both data simulations (i.e., multiple HIV-1 subtypes or CRFs) and available experimental sequencing data of HIV-1 and other viruses.

Among the *de novo* assemblers, Strainline provides satisfactory assemblies within a reasonable timeframe but requires a large amount of available memory, typically 64GB of RAM or more, exceeding the capacity of a standard computer. MetaFlye, though slower, is suitable for metagenomic sequencing data. GoldRush, the fastest assembler, lacks the capability to assemble alternative contigs and is thus unsuitable for metagenomic sequencing data. Canu efficiently allocates appropriate CPU and memory to each process, resulting in efficient CPU utilization and low memory consumption. Among the reference-based assemblers, iGDA and RVHaplo exhibit similar computational resource usage, with iGDA’s runtime comparable to Canu and GoldRush, and RVHaplo’s runtime comparable to Strainline. HaploDMF demonstrates considerably longer runtime due to heavy CPU usage during the assembly process, particularly in deep matrix factorization. However, utilizing a compatible GPU could mitigate both the runtime and CPU workload of HaploDMF. ^43^^,^^46^ Overall, the variations in computational performance among the assemblers emphasize the critical importance of careful assembler selection for the success of analyses tailored to specific scientific projects.

To improve HIV-1 quasispecies profiling and genomic surveillance, it’s essential to discuss the advantages and disadvantages of selected assemblers for handling heterogeneous viral or microbial metagenome sequencing data. Canu and GoldRush are non-strain aware *de novo* assemblers, lacking the ability to distinguish strains within a sample. Using a reference genome may overcome this limitation, but it may not be suitable for genomic surveillance of emerging infectious diseases. ^80^ MetaFlye is suitable for metagenomic assemblies, employing local k-mer distributions to establish a threshold for global k-mer counting, thus retaining less abundant sequencing reads. ^42^ Strainline is designed for haplotype reconstruction of diverse viral genomes, treating clusters of long reads as unique scaffolds of different haplotypes and extending scaffolds through multiple iterations of the Overlap-Layout-Consensus (OLC) algorithm. ^28^ However, this approach may be ineffective with equally long and highly similar reads, resulting in inaccurate estimates of haplotype numbers. For instance, Strainline has been observed to produce numerous duplicate contigs from HIV-1 group M subtype B from 2M HIV-1 mixture and 1M1C HIV-1 mixture.

High-quality reference sequences are crucial for reference-based assembly. The assemblers use a probabilistic approach to align reads to the reference sequence and identify variants like single nucleotide variants (SNVs), insertions, and deletions for haplotype phasing. Both RVHaplo and HaploDMF employ an SNV frequency matrix for clustering reads of different haplotypes. However, RVHaplo relies on overlapping SNV sites, which may be inadequate for assembling closely related genomes. In contrast, HaploDMF uses a deep matrix factorization model with an adapted loss function to learn and correct latent features from each iteration of SNV detection, ^43^ enhancing robustness against noise during SNV detection and enabling assembly of closely related strains. iGDA utilizes Adaptive-Nearest Neighbor clustering (ANN) to estimate the number of clusters and, like RVHaplo, uses overlapping SNVs and coverage for contig extension. ^44^ In general, reference-based assemblers seem suitable only for quasispecies profiling.

Since the choice of assembler significantly impacts genome assemblies, it is crucial to meet specific hardware system requirements tailored to match the quality of input data, genome sizes of the organisms of interest, and the algorithms used for genome assembly. However, the suitable system requirements have not been explicitly provided. Based on this benchmarking study, the following optimal computational requirements are suggested:

• All assemblers should be executed in a Linux-based operating system.
• A quad-core (4 cores) or higher CPU with x86 microarchitecture is recommended. CPUs with ARM-based microarchitecture (e.g., Apple Silicon) may require specific compiling or hand-tuned code with ARM instructions. Alternatively, a properly optimized code can achieve performance comparable to native x86 systems through dynamic binary translation (e.g., Rosetta). ^81^
• An 8 GB memory is sufficient for either viral or bacterial genome assembly, while a 16 GB RAM or higher is recommended for larger genomes, such as human or other mammals. It’s worth noting that this study utilized 96 GB memory for all assemblers.

An additional consideration is the balance between CPU and memory, as demonstrated by comparing a workstation with a generic home PC. An interesting finding is that despite both systems having the same number of CPU cores, the home PC took slightly longer, and its CPUs were more overutilized than those on the workstation. This disparity is attributed to the home PC needing to perform memory management more frequently, thereby adding load to the CPU and slowing down the process. ^82^

In addition, the impact of virtualization and containerization layering during benchmarking was disregarded. Consequently, the wall clock time measured from a server was longer than that measured from a workstation desktop. It is possible that the software spends more time traversing layers of virtualization instead of running on a bare-metal environment (i.e., installed the software directly on the machine without containerization). ^83^^–^^85^ Also worth mentioning, from a computer science perspective, it is expected that when evaluating the efficiency of algorithms used by each assembler, the concept of computational complexity should be applied to provide a better estimation of computational resource requirements and runtime on a more diverse set of data i.e. predicting computing time of dataset with different depth of coverage, read length and assembly complexity. ^79^^,^^86^^,^^87^ While acknowledging this consideration, it is important to note that conducting a benchmark on real machines provides practical insights and actionable data on a specific use case, despite potential confounders introduced by the machine itself or from software virtualization.

Insights on computational requirements for current long-read assemblers offer guidance for implementing edge computing in molecular surveillance and epidemiology in bioinformatics and virology. Long-read sequencing data of HIV-1 used for benchmarking indicates that a containerized HIV-64148 pipeline can function with sub-optimal computational infrastructures like workstation PCs or generic home PCs. This pipeline has the potential to support and expand HIV-1 surveillance networks, benefiting scientists, epidemiologists, healthcare providers, and people living with HIV.

### Ethical considerations

Not applicable.

## Data Availability

Figshare: HIV64148 TABLE_S6_HIV_Combinations_DataSimulation https://doi.org/10.6084/m9.figshare.25436023 ^54^ all accession numbers are provided in Table S6 Figshare: HIV64148 Template FASTA. https://doi.org/10.6084/m9.figshare.25435774 ^59^ This archive contains reference sequences in FASTA format used as templates to simulated Oxford Nanopore reads Each template is located within a subfolder corresponding to its simulation number. Figshare: HIV64148 Simulated FASTQ. https://doi.org/10.6084/m9.figshare.25435822 ^60^ This archive contains simulated reads from NanoSim with template FASTA. Each FASTQ is located within a subfolder corresponding to its simulation number. Figshare: HIV64148 QC Simulated FASTQ. https://doi.org/10.6084/m9.figshare.25435912 ^61^ This archive contains output from NanoPlot describing characteristic of Simulated FASTQ files. Each HTML is located within a subfolder corresponding to its simulation number. Figshare: HIV64148 Results. https://doi.org/10.6084/m9.figshare.25435948 ^64^ This archive contains genome assembly in FASTA, BLAST result in CSV, and HIV64148 report in HTML from 2M, 2C, 1M1C, and 2M1C HIV-1 mixtures, as well as the results from resource usage overtime (CPU, Memory) measured on 3 computational platforms over 7 tools in CSV format. Data are available under the terms of the Creative Commons Attribution 4.0 International license (CC-BY 4.0). Figshare: HIV64148 Supplementary data. https://doi.org/10.6084/m9.figshare.25436023 ^54^ This project contains the following extended data: **
Figure S1.** The MetaQUAST assessment evaluated contigs from the assemblers processing four median read-length FASTQ inputs: ( **A**) 1,000-nt, ( **B**) 2,000-nt, ( **C**) 3,000-nt, and ( **D**) 4,000-nt ( Table 3). The x-axis displays the percentage sequence similarity obtained from a BLAST alignment with the corresponding reference genomes, while the y-axis represents the aligned length, indicating the longest continuous alignment between each contig and its reference genome. Dot sizes indicate the genome fraction (%Ref Aligned), calculated as the ratio of continuous aligned bases to the total reference genome length. Subplots above and beside each figure display histograms of the contig counts. Additionally, ( **E**) shows the averaged genome fraction (%) of contigs from different long-read assembler pipelines. The complete data are available in Extended data, Table S9. ^54^ **
Figure S2.** The average completeness of major HIV-1 open reading frames (ORFs) of the contigs generated from the assemblers analyzing FASTQ inputs of 4 median read lengths: ( **A**) 1,000-nt, ( **B**) 2,000-nt, ( **C**) 3,000-nt, or ( **D**) 4,000-nt. **
Figure S3.** Contig size distribution of all assemblers processing the simulated FASTQ inputs of the 4 HIV-1 mixtures. **
Table S1.** A list of experimental data and additional information. N/A: not applicable. **
Table S2**. A statistical summary of runtime measurements taken from an assembly phase of the pipelines. **
Table S3**. The memory usage and maximum CPU utilization of each assembler on three computational systems. The benchmark utilized 100 samples of two HIV-1 group M mixture with 2,000x coverage and 8,000-nt median read length. **
Table S4**. The genome statistics of contigs generated from ( **A**) Full genome laboratory isolate NL4-3, ( **B**) Mpox propagated in a CV-1 cell line and ( **C**) Mpox from pustular lesion, and ( **D**) SARS-CoV-2 data by the selected assemblers. **
Table S5**. The genome statistics of contigs generated from ( **A**) Polio virus, ( **B**) Hepatitis C virus (HCV), and ( **C**) ZIKA Virus (ZIKV) data by the selected assemblers. **
Table S6.** HIV Combinations for Data Simulation (Excel) **
Table S7.** Characteristics of Simulated Samples Readlength Experiment (Excel) **
Table S8.** Characteristics of Simulated Samples HIV mixture Experiment (Excel) **
Table S9.** An Assembly Evaluation of HIV-1 Read Length Experiment (Excel) **
Table S10.** An Assembly Evaluation of HIV-1 Subtype Mixtures Experiment (Excel) **
Table S11.** Subtypes Recall Rates (Excel) **
Table S12.** REGA Subtyping (Excel) **
Table S13.** 5’ Half Genomes of NL4-3 Alignment Statistics (Excel) **
Table S14.** Other Viruses Alignment Statistics (Excel) All high-resolution figures are available in https://doi.org/10.6084/m9.figshare.25436125.v1. ^88^ Data are available under the terms of the Creative Commons Attribution 4.0 International license (CC-BY 4.0).

## Software and code

Software and code for this workflow can be found on GitHub: https://github.com/STTLab/HIV-64148 (License: OSI approved open license software is under GPL-3.0 license.)

Archived code as at time of publication: https://doi.org/10.6084/m9.figshare.25436062 ^89^

Code is available under the terms of the Creative Commons Attribution 4.0 International license (CC-BY 4.0).
