# Supplementary material for: Easing genomic surveillance: A comprehensive performance evaluation of long-read assemblers across multi-strain mixture data of HIV-1 and Other pathogenic viruses for constructing a user-friendly bioinformatic pipeline
Source: F1000Res. 2024 May 31;13:556. [Version 1] doi: 10.12688/f1000research.149577.1 (PMC11231628; doi:10.12688/f1000research.149577.1)
Supplement: Peer review history

# Reviewer report: Reviewer response for version 1

**DOI:** 10.5256/f1000research.164057.r297759

**Published:** 2024-07-16

**Authors:** Sira Sriswasdi

**Affiliations:** 1 Computational Molecular Biology Group, Faculty of Medicine, Chulalongkorn University, Bangkok, Bangkok, Thailand; 2 Center for Artificial Intellience in Medicine, Faculty of Medicine, Chulalongkorn University, Bangkok, Bangkok, Thailand

This work presents a comprehensive evaluation of de novo and reference-based assembly tools for Nanopore sequencing of genome mixtures of HIV and other viruses. Focus was placed on both the ability of the tools to capture the true viral subtypes and the computational resource requirement. The impact of basic quality filters, such as the minimum read length, was also explored. The best performing tools were selected based on simulated datasets of known HIV mixtures and their performance were evaluated on various actual and simulated datasets of multiple viruses. This work also provides a container resource that would be useful for non-experts to analyze their own data. However, there are some missing details and metrics that should be improved.

 Major issues:

1. Since a key limitation of Nanopore sequencing is the relatively high sequencing error (compared to other sequencing techniques), I think it is unrealistic to simulate the data with the --perfect mode which produces error-free reads. Furthermore, using error-free simulated data would underestimate the number of mismatches and overestimate the % similarity and correctness of the resulting contigs.
2. On the same issue of sequencing error, I wonder if more attention should be placed on optimizing the polishing step (such as Luan T, et al., (2024) [Ref-1]). In the current work, only the default error handling algorithm associated with each tool was used.
3. When evaluating the assembly characteristics of a mixture of subtypes, such as in Figure 6-8, and Figure S3, I think it is important to show the characteristics separately for each subtype so that it is clear whether all subtypes were assembled equally well. Or at least, please show the ranges of the values, not just the averages.
4. In Table 5, I’m not sure how a Spearman correlation could be calculated with assembler selection, which is not an ordinal variable.
5. When evaluating the assemblers using simulated mixture data, it is unclear what proportion of reads coming from each subtype was used. I think the impact of fractional abundance on the quality of the assembled genomes should be investigated. For the simulated HIV-1 mixture datasets in Table 3, please consider exploring different mixture ratios. For the pre-built simulated polio, zika, and HCV datasets, please indicate the mixture ratios to aid interpretation.
6. There is no apparent tuning of the parameters for the assemblers. Since this is a benchmark paper that releases a container that will be used by the community, please consider more optimization of the pipeline.

Minor issues:

1. The comparison between de novo assemblers and reference-based assemblers needs to be carefully explained because reference-based approaches, with reference genome as prior knowledge, would in general be able to produce longer contigs (as seen in Figure 5D-F versus Figure 5A-C, with Figure 5F as the extreme case where every contig from RVHaplo is almost the entire genome). It would be nice to explain these to the readers to prevent misinterpretation.
2. Figures 10 and 11 could be improved by annotating each contig with the identity of each viral subtype in the mixture. This would allow the readers to understand (i) whether the correct contigs correspond to individual subtypes, (ii) which subtypes could not be recovered, and (iii) whether different methods misassemble the same subtypes.
3. The pipeline seems to use a fixed HIV-1 reference NC_001802.1. Should the impact of the reference genome be explored? Should the reference genome be changeable in the released pipeline?

Is the work clearly and accurately presented and does it cite the current literature?

Yes

If applicable, is the statistical analysis and its interpretation appropriate?

Partly

Are all the source data underlying the results available to ensure full reproducibility?

Yes

Is the study design appropriate and is the work technically sound?

Partly

Are the conclusions drawn adequately supported by the results?

Yes

Are sufficient details of methods and analysis provided to allow replication by others?

Yes

Reviewer Expertise:

molecular evolution of microorganisms, cancer transcriptomics, bioinformatics pipelines, machine learning and deep learning applications in biomedicine

I confirm that I have read this submission and believe that I have an appropriate level of expertise to confirm that it is of an acceptable scientific standard, however I have significant reservations, as outlined above.

## References

[1] : Benchmarking short and long read polishing tools for nanopore assemblies: achieving near-perfect genomes for outbreak isolates. *BMC Genomics* .2024;25(1) : 10.1186/s12864-024-10582-x 679 10.1186/s12864-024-10582-x 38978005 PMC11232133

# Reviewer report: Reviewer response for version 1

**DOI:** 10.5256/f1000research.164057.r297758

**Published:** 2024-07-08

**Authors:** Rujira Chaysiri

**Affiliations:** 1 School of Management Technology, Sirindhorn International Institute of Technology, Thammasat University, Pathum Thani, Thailand

The study conducted a comprehensive evaluation of seven genome assemblers, focusing on their performance across different computational platforms including a server, workstation PC, and standard PC. It was found that while the choice of platform did not significantly affect processing time, memory capacity was a critical factor, with some assemblers like Strainline requiring up to 64 GB of memory, rendering them unsuitable for standard PCs. Reference-based assemblers were generally faster and more consistent in their processing times compared to the variable performance of de novo assemblers. When assessing the minimum read lengths necessary for effective assembly, de novo assemblers needed a minimum read length (N50) of at least 2000 nt to function optimally, while reference-based assemblers were less affected by variations in read length and consistently produced assemblies close to the expected genome size of 8000 nt for HIV.

 In evaluating the performance on mixed HIV-1 subtypes, de novo assemblers exhibited lower recovery rates for subtypes, indicating challenges in reconstructing all subtypes present in the input. Conversely, reference-based assemblers demonstrated better recovery of subtypes but tended to over-represent subtype B, used as the reference, and had higher rates of indels, pointing to a need for improved error correction. Further testing on real sequencing data from various viruses, including HIV-1, Mpox, SARS-CoV-2, Polio, Hepatitis C, and ZIKV, showcased the versatility and high-quality results of the selected assemblers across different viral genomes.

 Additionally, the study detailed the development of a Docker container for the HIV-64148 pipeline, which incorporated the tested assemblers along with features for querying online databases to generate comprehensive reports on subtype composition, mutations, and drug resistance profiles. This containerized pipeline proved to be practical and accessible, capable of operating on both large servers and standard computers, thus enhancing its utility for genomic surveillance tasks in diverse settings.

 The study provides a robust evaluation of genome assemblers, delivering valuable insights into their performance across different computational platforms and data sets. Its comprehensive nature, covering multiple aspects of assembler performance such as processing time, memory usage, and accuracy, is a significant strength. The inclusion of both simulated and real sequencing data from a variety of viruses highlights the practical applicability of the findings. Additionally, the development of a Docker container for the HIV-64148 pipeline enhances accessibility and usability, facilitating standardized genomic surveillance.

 However, the study also reveals some limitations. The high memory requirements for some assemblers, particularly Strainline, limit their usability on standard PCs, which could restrict their application in resource-limited environments. The tendency of reference-based assemblers to over-represent certain subtypes, such as subtype B in HIV, introduces bias that could affect the reliability of genomic studies. Furthermore, the higher indel rates observed in reference-based assemblies indicate a need for further refinement and error correction to improve the accuracy of the results.

 To address these issues, future research should focus on optimizing memory usage for assemblers, making them more accessible on standard hardware. Developing methods to reduce reference bias in reference-based assemblers would enhance the reliability of their results. Additionally, implementing robust error correction algorithms could mitigate the higher indel rates observed in reference-based assemblies. Overall, while the study makes significant contributions to the field of genomics, addressing these limitations would further improve the effectiveness and applicability of genome assemblers.

Is the work clearly and accurately presented and does it cite the current literature?

Yes

If applicable, is the statistical analysis and its interpretation appropriate?

Yes

Are all the source data underlying the results available to ensure full reproducibility?

Yes

Is the study design appropriate and is the work technically sound?

Yes

Are the conclusions drawn adequately supported by the results?

Yes

Are sufficient details of methods and analysis provided to allow replication by others?

Yes

Reviewer Expertise:

Management Technology

I confirm that I have read this submission and believe that I have an appropriate level of expertise to confirm that it is of an acceptable scientific standard.

# Reviewer report: Reviewer response for version 1

**DOI:** 10.5256/f1000research.164057.r297757

**Published:** 2024-07-05

**Authors:** Udom Sae-Ueng

**Affiliations:** 1 National Center for Genetic Engineering and Biotechnology (BIOTEC), National Science and Technology Development Agency (NSTDA), Pathum Thani, Thailand

1. The title of the paper should focus on the performance of genome assemblers, as the selection of the assembler is the main factor in the pipeline.

 2. A significant number of mismatch errors occur in ONT sequencing data. The paper should address how to ensure that these errors will assemble into distinct vital haplotypes. It should also explain the dissimilarities between viral haplotypes in genomes and provide evidence of low-abundance haplotypes with read ratios in real-world situations for quasispecies analysis.

 3. If the ratio of reads used in the HIV mixture is the same (as shown in Table 3), but different genome assemblers are used (as shown in Figure 9), it results in different subtype recalls. This could be considered a bias of genome assemblers.

Is the work clearly and accurately presented and does it cite the current literature?

Yes

If applicable, is the statistical analysis and its interpretation appropriate?

Yes

Are all the source data underlying the results available to ensure full reproducibility?

Yes

Is the study design appropriate and is the work technically sound?

Yes

Are the conclusions drawn adequately supported by the results?

Yes

Are sufficient details of methods and analysis provided to allow replication by others?

Yes

Reviewer Expertise:

Genome sequencing; Biotechnology

I confirm that I have read this submission and believe that I have an appropriate level of expertise to confirm that it is of an acceptable scientific standard.
